# DNA Methylation and RNA-DNA Hybrids Regulate the Single-Molecule Localization of a DNA Methyltransferase on the Bacterial Nucleoid

**DOI:** 10.1128/mbio.03185-22

**Published:** 2023-01-16

**Authors:** Nicolas L. Fernandez, Ziyuan Chen, David E. H. Fuller, Lieke A. van Gijtenbeek, Taylor M. Nye, Julie S. Biteen, Lyle A. Simmons

**Affiliations:** a Department of Molecular, Cellular, and Developmental Biology, University of Michigan, Ann Arbor, Michigan, USA; b Department of Biophysics, University of Michigan, Ann Arbor, Michigan, USA; c Department of Chemistry, University of Michigan, Ann Arbor, Michigan, USA; University of Chicago

**Keywords:** replisome, epigenetic, superresolution microscopy, *Bacillus subtilis*, restriction modification

## Abstract

Bacterial DNA methyltransferases (MTases) function in restriction modification systems, cell cycle control, and the regulation of gene expression. DnmA is a recently described DNA MTase that forms N6-methyladenosine at nonpalindromic 5′-GACGAG-3′ sites in Bacillus subtilis, yet how DnmA activity is regulated is unknown. To address DnmA regulation, we tested substrate binding *in vitro* and found that DnmA binds poorly to methylated DNA and to an RNA-DNA hybrid with the DNA recognition sequence. Further, DnmA variants with amino acid substitutions that disrupt cognate sequence recognition or catalysis also bind poorly to DNA. Using superresolution fluorescence microscopy and single-molecule tracking of DnmA-PAmCherry, we characterized the subcellular DnmA diffusion and detected its preferential localization to the replisome region and the nucleoid. Under conditions where the chromosome is highly methylated, upon RNA-DNA hybrid accumulation, or with a DnmA variant with severely limited DNA binding activity, DnmA is excluded from the nucleoid, demonstrating that prior methylation or accumulation of RNA-DNA hybrids regulates the association of DnmA with the chromosome *in vivo*. Furthermore, despite the high percentage of methylated recognition sites and the proximity to putative endonuclease genes conserved across bacterial species, we find that DnmA fails to protect B. subtilis against phage predation, suggesting that DnmA is functionally an orphan MTase involved in regulating gene expression. Our work explores the regulation of a bacterial DNA MTase and identifies prior methylation and RNA-DNA hybrids as regulators of MTase localization. These MTase regulatory features could be common across biology.

## INTRODUCTION

Restriction modification (RM) systems were one of the first recognized defense mechanisms that bacteria use to thwart bacteriophage infection (1, 2). Initial bacteriophage studies identified that only phage that have been modified by a host can successfully infect the host (2). This modification was later identified as DNA methylation from enzymes called DNA methyltransferases (MTases) (reviewed in references 3 and 4). MTases from RM systems modify DNA by adding a methyl group in a sequence-specific context to form either N6-methyladenosine (m6A), N4-methylcytosine, or 5-methylcytosine (5). Genes encoding MTase function are often adjacent to genes encoding restriction endonuclease (REase) activity (6). If a cell encodes an RM system and unmethylated DNA enters the host cell, for example, from a phage, REase will degrade the invading DNA before it can be replicated (6).

In addition to functioning in RM systems, DNA methylation regulates other processes, including DNA replication, DNA repair, and transcription (7). Many Gammaproteobacteria encode *dam*, which is referred to as an orphan DNA MTase because it lacks a cognate REase enzyme (3, 7, 8). In Escherichia coli, DNA methylation by Dam influences the timing of replication and aids in the excision of mismatched bases from the new DNA strand following replication during methyl-directed mismatch repair (3). Alphaproteobacteria also encode the conserved orphan MTase *ccrM*, which regulates the timing of DNA replication and is essential for Caulobacter crescentus growth in rich media (3).

Epigenetic regulation of gene expression in bacteria results from the interaction between certain DNA-binding proteins and methylated DNA (9). Orphan and RM-associated MTase enzymes influence gene expression and bacterial behaviors through DNA methylation, with examples ranging from pili expression in E. coli, eukaryotic cell adhesion in Campylobacter jejuni, and virulence regulation in Streptococcus pyogenes (10–12). Studies have benefited from the use of single-molecule real-time (SMRT) sequencing analysis to characterize the methylome and identify sites of methylation followed by predicting the MTase enzymes responsible for the corresponding modification (8).

Previously, we used SMRT sequencing to characterize the methylome of the Gram-positive soil bacterium Bacillus subtilis (13). We identified the DNA MTase DnmA (M.BsuPY79I), which recognizes the six-base-pair, nonpalindromic sequence 5′-GACG**A**G-3′ and methylates adenine to form m6A (13). *In vitro* methylation assays with DnmA demonstrated substrate specificity: double-stranded DNA (dsDNA) harboring the methylation site was identified as the optimal substrate, followed by single-stranded DNA (ssDNA) and ssRNA (13). DNA substrate compositions heavily influence DNA and MTase interactions *in vitro* for some well-characterized MTases, but how these *in vitro* experiments inform *in vivo* activity is not well understood (14, 15). The *dnmA* gene is flanked by *yeeB* and *yeeC*, two genes with putative REase functions, in a genetic structure suggestive of an operon from a horizontally acquired element. While deletion of *dnmA* alters the expression of a subset of genes, the growth rate and restriction of plasmid uptake are unchanged. Therefore, it remains unclear if *dnmA-yeeB-yeeC* are functional under stress conditions, such as bacteriophage infection.

In this study, we identify how different substrates influence the *in vitro* DnmA binding kinetics and how that affects *in vivo* DnmA dynamics. We also investigate the conservation of the gene synteny and architecture between *dnmA* and its genetic neighbors across many bacterial species, and we assess the role of *dnmA* in response to bacteriophage infection. We show that the association of DnmA with DNA *in vitro* and *in vivo* is regulated by prior DNA methylation and formation of RNA-DNA hybrids. We also show that DnmA searches the entire nucleoid but localizes more strongly at the replisome position, suggesting that binding site recognition can occur anywhere on the chromosome with preference for positions near the replisome. Furthermore, we find that *dnmA* and the flanking genes *yeeB* and *yeeC* do not function as an active RM system and fail to protect B. subtilis from phage predation. Our work demonstrates how substrate specificity alters the *in vivo* localization of an MTase that arises from a restriction modification relic, causing DnmA to function as an orphan MTase in the regulation of gene expression in B. subtilis.

## RESULTS

### Localization of DnmA-PAmCherry *in vivo*.

**FIG 1:**
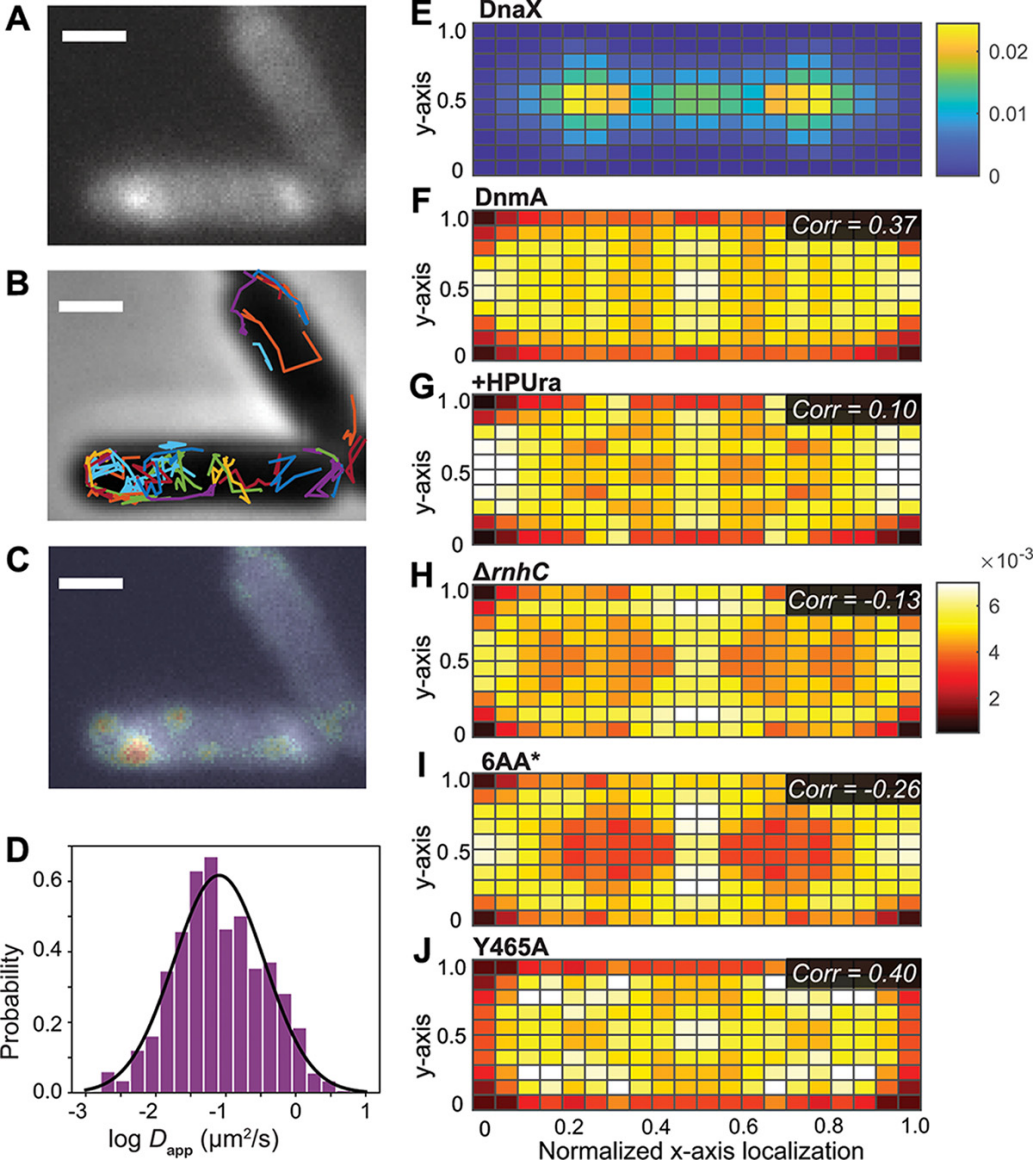
Single-molecule characterization of *in vivo* DnmA dynamics and localization. (A) Fluorescence image of DnaX-mCitrine. Scale bars = 1 μm for panels A to C. (B) False-colored single-molecule trajectories of DnmA-PAmCherry in two representative WT cells overlaid on the phase-contrast image of the B. subtilis cells. (C) Overlay of single-molecule localizations of DnmA-PAmCherry (jet heatmap) and fluorescence image of DnaX-mCitrine (grayscale). (D) Normalized histogram showing the distribution of the log diffusion coefficients of the single-molecule trajectories of DnmA-PAmCherry. Black line, Gaussian fit to the log diffusion coefficient distribution. The histogram and fit curve are weighted by track length. (E to J) Normalized localization probability density maps of (E) DnaX-mCitrine, (F) WT DnmA-PAmCherry, (G) +HPUra DnmA-PAmCherry, (H) Δ*rnhC* DnmA-PAmCherry, (I) DnmA[6AA*]-PAmCherry, and (J) DnmA[Y465A]-PAmCherry, all within a normalized cell. Single-molecule localizations are projected along the long and short axes of the cell, normalized to their relative position, and resymmetrized along the axes. Colormaps show localization probability. *Corr* in panels F to J, Pearson’s correlation of that DnmA variant’s localization heatmap with the DnaX localization heatmap. Each single-molecule data set was acquired from 4 distinct days from independent cultures.

Our prior work showed that DnmA is both necessary and sufficient to methylate dsDNA *in vitro* and *in vivo* (13). Given the role of DnmA in altering gene expression (13), it is important to understand how DnmA interacts with DNA *in vivo*. To this end, we generated a B. subtilis strain in which the wild-type (WT) *dnmA* allele was replaced with a gene encoding DnmA fused to a photoactivatable fluorescent protein, PAmCherry, at the C terminus (*dnmA-PAmCherry*). To ensure that DnmA-PAmCherry retained methyltransferase activity *in vivo*, we measured the activity of a transcriptional reporter that is dependent on DnmA (13). We found that reporter activity is the same between WT and DnmA-PAmCherry (see Fig. S1A in the supplemental material), indicating that the C-terminal tag does not interfere with DnmA function. Further, Western blot analysis demonstrated that the DnmA-PAmCherry fusion is not degraded *in vivo* (Fig. S1B). Based on photoactivation and tracking of single copies of DnmA-PAmCherry in living cells (Materials and Methods) (Fig. 1B) (16), we observed the localization of this protein in its native environment in *N *= 1,766 single-molecule trajectories in *n *= 275 B. subtilis cells growing exponentially in defined minimal medium. We categorized the motion of these molecules based on fitting each single-molecule trajectory to a linear mean-square displacement model for normal diffusion (Materials and Methods); the histogram of the log diffusion coefficients for DnmA-PAmCherry trajectories weighted by the track length is given in Fig. 1D. As a positional reference for nascent DNA, we imaged fusions of the replisome component DnaX to the fluorescent protein mCitrine in a separate fluorescence channel (Fig. 1A).

10.1128/mbio.03185-22.1 FIG S1 DnmA-PAmCherry variant stability and fusion activity *in vivo*. (A) Western blot analysis of DnmA-PAmCherry variants. The black arrow points to DnmA-PAmCherry. (B) Flow cytometry analysis of a green fluorescent protein GFP transcriptional reporter that is regulated by DnmA (*amyE*::*P_scpA_-GFP*) (13). White bars indicate either WT DnmA or *dnmA* deletion, while filled bars indicate either WT DnmA, Y465A DnmA, or 6AA DnmA fused to PAmCherry in an otherwise WT background. Bars represent the mean from six biological replicates (gray filled circles), and error bars represent the standard deviation. Asterisks indicate statistical significance (*P* < 0.05) compared to the WT background using the Wilcox test. Download FIG S1, EPS file, 1.7 MB. Copyright © 2023 Fernandez et al. 2023 Fernandez et al. https://creativecommons.org/licenses/by/4.0/ This content is distributed under the terms of the Creative Commons Attribution 4.0 International license.

The overlay of the superresolution images of DnaX-mCitrine (grayscale) and DnmA-PAmCherry (jet) shows some spatial overlap for DnmA and DnaX, although the DnmA positions are more spread out over the region of the cell occupied by the nucleoid (Fig. 1C). To further quantify their spatial correlation at the population level, we generated a normalized localization density map of DnmA to determine the localization pattern of DnmA in 275 WT cells (Fig. 1F). We also generated a normalized localization density map of the replisome by analyzing DnaX-mCitrine (Fig. 1E) (17). The Pearson correlation between the two heatmaps is 0.37, showing that DnmA has a positive spatial correlation with the replisome. Due to the nonpalindromic nature of the DnmA recognition site, nascent DNA will be unmethylated postreplication, acting as a substrate for methylation by DnmA. Thus, our data suggest that binding and methylation of nascent, unmethylated DNA drive the correlative positioning of DnmA and DnaX, although DnmA does explore much more of the nucleoid region in the cell.

### Manipulating available substrate *in vivo* disrupts DnmA localization.

**FIG 2:**
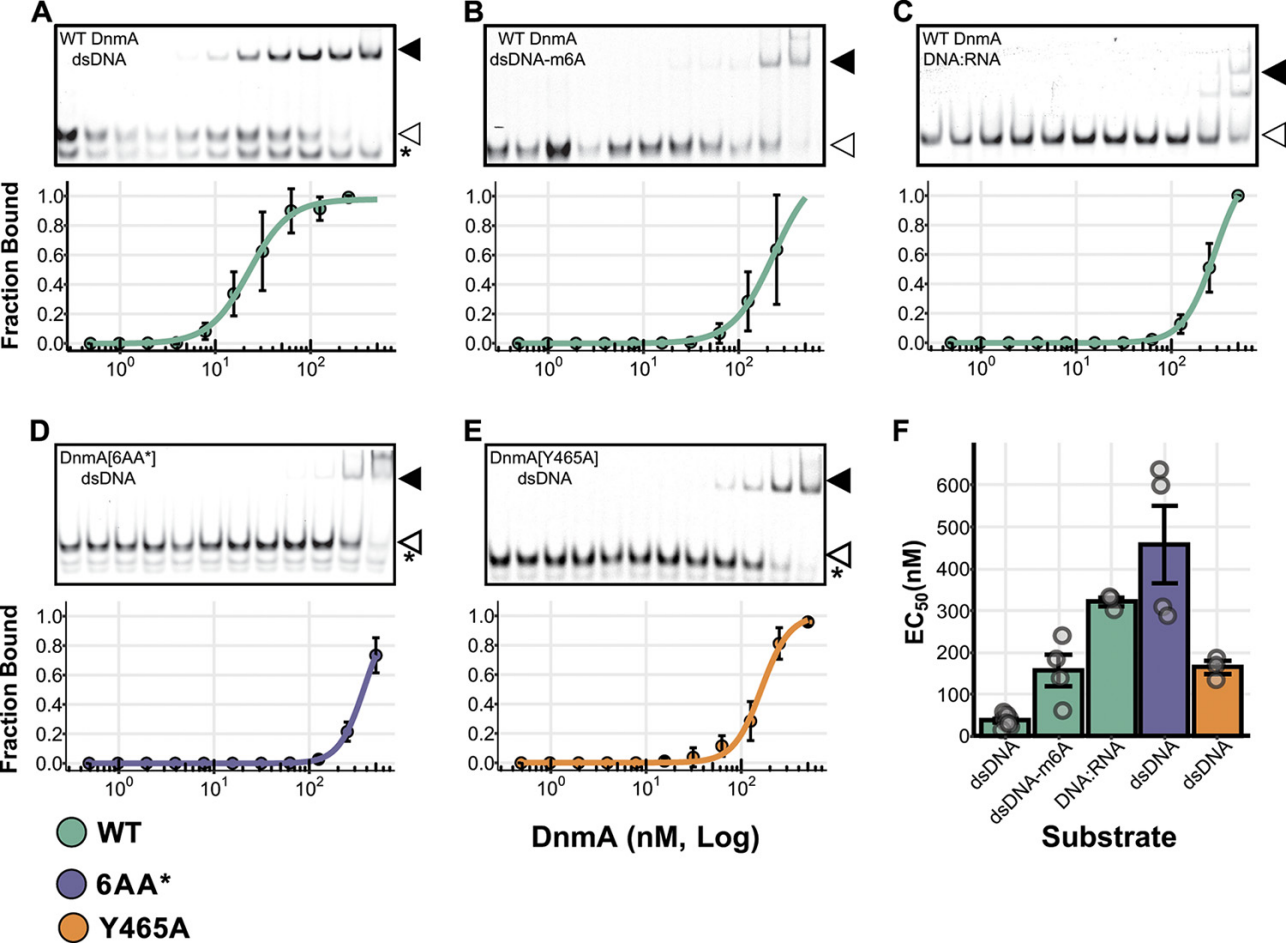
Substrate and key residues important for DNA binding and catalysis influence DNA interactions *in vitro*. (A to E) EMSA experiments with DnmA variants and different DNA substrates. Representative gels showing unshifted bands (white arrows), shifted bands (black arrows), or unannealed single-stranded DNA (asterisks) (top) and quantitation of fraction bound with increasing concentrations of DnmA (bottom), where points represent the average, error bars represent standard deviations, and lines are modeled from four-parameter log-logistic equations. DnmA variant and DNA substrate are in the top-left corner of the representative gel. (F) Average ± standard deviation of estimated half maximal concentrations (EC_50_) for DNA binding calculated from the binding curves. Points represent individual experiments, and bar fill colors represent the DnmA variant.

DNA binding is heavily influenced by substrate, where most N6-DNA MTase enzymes tend to have lower binding affinities toward substrates that are not dsDNA *in vitro* (14, 15). We hypothesized that the position of DnmA can be explained by the availability of unmethylated substrate near the replisome, where unmethylated dsDNA would be enriched shortly after DNA replication. To test this hypothesis, we first set out to establish how altering DNA substrate influences DnmA binding *in vitro* using electrophoretic mobility shift assays (EMSAs). In addition to unmethylated dsDNA substrate, we utilized methylated dsDNA and an RNA-DNA hybrid as candidate substrates for possible *in vivo* DNA modifications or perturbations. Methylated dsDNA is the primary DNA species in B. subtilis grown under standard conditions, while RNA-DNA hybrids are transiently found throughout the genome from DNA replication and highly transcribed regions (13, 18). DnmA binds to unmethylated dsDNA with the greatest estimated affinity (50% effective concentration [EC_50_], 36.8 ± 14.2 nM [mean ± standard deviation [SD]) and has much lower estimated affinities for methylated dsDNA and RNA-DNA hybrids (EC_50_, 156.4 ± 76.0 nM and 321.4 ± 16.5 nM, respectively; Fig. 2A, C, and F). Though the range of DnmA concentrations for the methylated dsDNA and RNA-DNA substrates makes affinity calculations less accurate, we conclude that DnmA binds preferentially to unmethylated dsDNA relative to methylated dsDNA or RNA-DNA hybrids *in vitro*.

Since DnmA binds to unmethylated dsDNA with the greatest affinity *in vitro*, we reasoned that changing the pool of this substrate *in vivo* would alter DnmA dynamics and localization *in vivo*. We measured DnmA-PAmCherry localization after treating B. subtilis with the replication inhibitor 6-(*p*-hydroxyphenylazo)-uracil (HPUra), which depletes the pool of available unmethylated dsDNA substrate in the cell (19). We acquired single-molecule tracking data from *N = *1,047 trajectories within *n *= 233 cells. We found that HPUra treatment decreases the average diffusion coefficient (Fig. S2A). The decreased affinity for methylated dsDNA *in vitro* suggests that the weight fraction of slow-moving DnmA molecules in HPUra-treated cells should decrease. However, we observe a slight increase in the weight fraction of slow-moving molecules in HPUra-treated cells compared to untreated cells with a concomitant decrease in the weight fraction of fast-moving molecules (Fig. S2G and H). We also found that the DnmA correlation with DnaX decreases from 0.37 to 0.10 in HPUra-treated cells (Fig. 1G). These data suggest that HPUra treatment likely does not decrease DNA binding throughout the nucleoid but does negatively influence DNA binding near the replisome. Of note, this marked change in the DnmA-DnaX spatial correlation is observed *in vivo* even though 99.7% of DnmA recognition sites are methylated during exponential growth (13).

10.1128/mbio.03185-22.2 FIG S2 Single-molecule tracking analysis. (A) Single-component Gaussian fit to the log diffusion coefficient distribution. The fit curves are weighted by track length. (B to F) Normalized histogram showing the distribution of the log diffusion coefficients of the single-molecule trajectories of DnmA-PAmCherry. Black dashed line, two-component Gaussian fit to the histogram; blue line, fit of the slower component; red line, fit of the faster component. *F*_bound_ indicates the weight fraction for the slower component in each fit. The histograms are weighted by track length. (G and H) Two-state (G) and three-state (H) weight fraction bar plots from Spot-On analysis (41) of the five DnmA single-molecule tracking data sets. In the Spot-On fitting, the average diffusion coefficient for each component is kept within the confidence interval of the WT diffusion coefficient to directly compare weight fractions (see Materials and Methods). Download FIG S2, EPS file, 2.9 MB. Copyright © 2023 Fernandez et al. 2023 Fernandez et al. https://creativecommons.org/licenses/by/4.0/ This content is distributed under the terms of the Creative Commons Attribution 4.0 International license.

Next, we measured DnmA-PAmCherry localization in B. subtilis cells lacking the RNase HIII gene *rnhC*, which is suggested to remove RNA-DNA hybrids in the genome (18). We acquired the Δ*rnhC* single-molecule tracking data from *N = *1,348 trajectories within *n *= 226 Δ*rnhC* B. subtilis cells. Unlike the WT cells, in which DnmA and DnaX are positively spatially correlated, the localization density map of DnmA in Δ*rnhC* cells has a negative spatial correlation with DnaX (−0.13; Fig. 1H). Further, more of the DnmA-PAmCherry molecules move slowly in Δ*rnhC* than in the WT (~50% slow population for WT compared to ~60% for Δ*rnhC*; Fig. S2G). In summary, this mutation has a marked effect in decreasing the colocalization of DnmA with DnaX and causes a subtly reduced average diffusion coefficient, resulting in an increase in the fraction of molecules diffusing slowly.

### The DNA binding variant DnmA[6AA*] localizes away from the replisome and the nucleoid.

Our data suggest that DNA binding and methylation explain DnmA-PAmCherry localization *in vivo*. To test this hypothesis, we generated variants of DnmA with amino acid substitutions at key residues involved in DNA binding and catalysis. DnmA is 57% similar to MmeI, a type II DNA MTase for which a structure is available (20). We structurally aligned DnmA with MmeI and identified putative residues important for DnmA interaction with its cognate sequence. Interestingly, single alanine substitutions in MmeI or other methyltransferases are often unable to completely abrogate DNA binding *in vitro* and can sometimes cause recognition of a different sequence (20–22), likely due to the high number of contacts between the residues in the DNA binding pocket and DNA (Fig. S3). Therefore, we designed a six-amino acid alanine substitution variant of DnmA (DnmA[6AA*]) which has substitutions at key residues we predict are involved in 5′-GACGAG-3′ recognition (Fig. S3). Further, we generated a catalytically inactive DnmA variant by introducing an alanine at position 465, replacing a tyrosine needed for stabilizing base-flipping during the methyl transfer reaction (DnmA[Y465A]), reviewed in reference (14). We have previously shown that this substitution renders DnmA inactive *in vivo* and *in vitro* (13). *In vitro* analysis of unmethylated dsDNA binding by the DnmA variants showed a decrease in estimated affinity to DNA, with the most severe effect in DnmA[6AA*], which had a 12-fold greater EC_50_ (458.3 ± 185.2 nM) than the WT DnmA, while DnmA[Y465A] had a 4-fold greater EC_50_ (164.2 ± 26.8 nM) (Fig. 2D to F).

10.1128/mbio.03185-22.3 FIG S3 Predicted DnmA residues involved in target sequence recognition and catalysis. Predicted interactions between DnmA and its target sequence 5′-GACGAG-3′. Residues that are biochemically similar to aligned residues in the homologous MTase MmeI are in boldface, while residues that are identical are in boldface italic. Residues chosen for alanine substitutions are in blue font. The gray shading surrounding 5′-CGA-3′ represents the shared nucleotides between the cognate recognition sites for DnmA and MmeI. Download FIG S3, PDF file, 0.6 MB. Copyright © 2023 Fernandez et al. 2023 Fernandez et al. https://creativecommons.org/licenses/by/4.0/ This content is distributed under the terms of the Creative Commons Attribution 4.0 International license.

We also introduced the DnmA variants fused to PAmCherry into the cell and checked for stability and functionality *in vivo*. The DnmA variants were not degraded *in vivo*, demonstrated by intact DnmA-PAmCherry fusions in Western blot analysis (Fig. S1A). Importantly, the variants were unable to complement reporter activity in a *ΔdnmA* background, indicating that both DnmA[6AA*] and DnmA[Y465A] are inactive *in vivo* (Fig. S1B). Single-molecule tracking data and normalized localization density maps were generated for these two variants. The diffusion coefficient distributions for the two variants are lower than those of WT DnmA-PAmCherry (Fig. S2A). The two variants demonstrated a decreased ability to bind DNA *in vitro*, yet *in vivo* we observed an increase in the weight fraction of slow-moving molecules (Y465A, 70%; 6AA*, 60%; Fig. S2G) compared to that of WT DnmA (50%; Fig. S2G). Strikingly, DnmA[6AA*] also has a strong negative correlation with DnaX (−0.26), while DnmA[Y465A] has a correlation similar to that of WT DnmA (0.40 versus 0.37; Fig. 1I and J). These data suggest that DnmA[Y465A] is still able to scan and search DNA for available substrate but is unable to catalyze methylation because of its inability to stabilize the flipped base, whereas the DnmA[6AA*] variant is unable to scan and search DNA, relegating it to positions outside the nucleoid region. Taken together, our results indicate that, regardless of substrate or variant, the mobility of DnmA is slower under these conditions and that DnmA localization is primarily influenced by DNA binding rather than by active methylation.

### DnmA is part of a conserved gene cluster with YeeB and YeeC.

**FIG 3:**
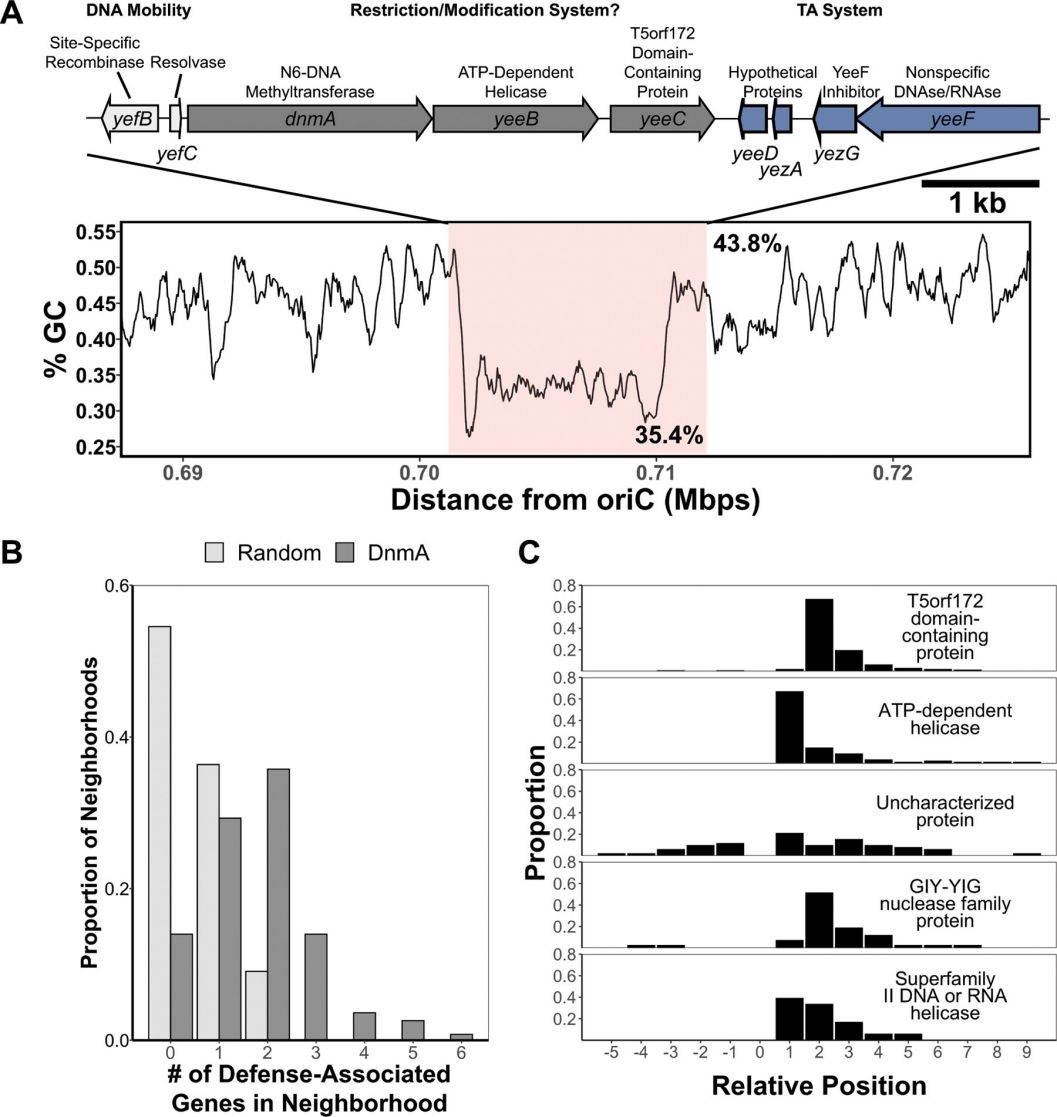
Neighborhood analysis of DnmA and DnmA homologs. (A) (Top) Genome architecture of the locus surrounding *dnmA* in B. subtilis PY79. (Bottom) Percent GC content of the B. subtilis PY79 genome approximately 15 kb upstream and downstream of the *dnmA* locus. The GC content from *yefB* to *yeeF* is highlighted in pink to emphasize the local minimum. The mean percent GC inside the pink box is 35.4%, and 43.8% is the mean percent GC of the genome. (B) The proportion of genome neighborhoods with a given number of defense-associated protein families. Light gray, the distribution from randomly sampled genomic neighborhoods; dark gray, the distribution from neighborhoods surrounding DnmA homologs. (C) The relative positions of the top five most frequent neighboring defense-associated protein families. 0 indicates the position of *dnmA*, positive integers indicate positions downstream (3′) of *dnmA*, and negative integers indicate positions upstream (5′) of *dnmA*.

Our *in vivo* single-molecule results suggest that DnmA, in part, colocalizes with the replisome to fully methylate the B. subtilis chromosome as replication occurs, raising questions about the function of m6A in B. subtilis. We have previously shown that m6A regulates the transcription of a subset of genes and that there is no difference in transformation efficiency in cells lacking m6A under the conditions tested (13). However, we had not tested a role for m6A in protection from bacteriophage predation. In prior work, we showed that m6A functions in the Gram-positive pathogen Streptococcus pyogenes both in the regulation of gene expression and as part of a functioning RM system, supporting the idea that DnmA can play a role in restriction modification as part of the putative operon consisting of *dnmA*, *yeeB*, and *yeeC* genes (12). YeeB has a C-terminal superfamily II DNA/RNA helicase domain like those found in restriction endonucleases, while YeeC has a C-terminal T5 orf172-domain, a largely uncharacterized domain that is predicted to have multiple functions involving DNA binding (23). In a bioinformatic survey, Makarova et al. identified YeeB and YeeC homologs as putative antiphage genes often found in a type of genomic island termed defense islands, suggesting that the *dnmA* operon could be involved in phage defense (24). The *dnmA* gene is also adjacent to two genes involved in DNA mobility (*yefB* and *yefC*) and to two putative toxin-antitoxin systems (*yeeD-yezA* and *yezG-*yeeF), while the whole region from *yefB* to *yeeF* is in a local GC-minimum compared to the surrounding genome (Fig. 3A). Together, these findings suggest that *dnmA*, *yeeB*, and *yeeC* were horizontally acquired and could represent a phage defense island (24, 25).

Given the information above, we asked if the *dnmA-yeeB-yeeC* gene cluster is conserved in other microorganisms and adjacent to genes with defense-associated protein families. We analyzed the genomic neighborhoods surrounding homologs of DnmA (10 genes upstream and 10 genes downstream) and scored the number of genes with predicted defense-associated protein families (see Materials and Methods). Neighborhoods harboring DnmA had, on average, 1.8 ± 1.2 genes with defense-associated protein families, while randomly selected regions of similar size had 0.56 ± 0.67 genes with defense-associated protein families (Fig. 3B). The most common protein families adjacent to *dnmA* were homologous to *yeeB* (ATP-dependent helicase/superfamily II DNA or RNA helicase protein families) and to *yeeC* (T5 orf172-domains containing protein/GIY-YIG nuclease protein families) (Fig. 3C). In addition, these protein families were found at the 1st and 2nd positions downstream of *dnmA*, respectively, indicating that the operon structure in these organisms is the same as the gene organization found in B. subtilis (Fig. 3C). The Uncharacterized protein family, which likely represents multiple protein functions, is found throughout the neighborhood upstream or downstream of *dnmA.* This family could represent another member of the *dnmA-yeeB-yeeC* locus in some bacteria; however, these genes are uncharacterized, with no known function, making their level of functional conservation unclear.

### The DnmA recognition motif is found in bacteriophage genomes.

The fact that YeeB and YeeC cooccur with DnmA in a conserved cluster and that YeeB and YeeC have putative antiphage activities suggests that the DnmA-YeeB-YeeC gene cluster functions as a restriction modification system. One antirestriction strategy by bacteriophage is the avoidance of a given restriction site within their genome, a phenomenon often observed for type II RM systems composed of one MTase and one REase (26). If the *dnmA-yeeB-yeeC* gene cluster functions as an RM system, then one prediction is that the DnmA recognition motif would be underenriched in bacteriophage genome sequences. We tested this hypothesis by comparing the observed number of recognition motifs to the expected number of recognition motifs in a sample of bacteriophage genomes, using observed-expected (O/E) ratios of 0.72 and 1.30 as thresholds for under and overenrichment, respectively (26). As a control, we measured the O/E ratio of the recognition sequence for the type II 5-methylcytosine MTase BsuMM (5′-CTCGAG-3′), which is part of an active type II RM system found in B. subtilis PY79 (27). In genomes with at least 5 expected motifs, the BsuMM motif has a mean O/E ratio of 0.43 (Fig. S4). Furthermore, 62.2% of the analyzed genomes have an O/E ratio below the threshold of 0.72, indicating the BsuMM motif is underenriched in bacteriophage genomes. We repeated the same analysis with the DnmA recognition motif 5′-GACGAG-3′ and a mock recognition motif with the same GC content as the DnmA recognition motif (5′-CTGCTC-3′). In contrast to the BsuMM motif, the DnmA and mock DnmA motifs have O/E ratios of 0.97 and 0.99, respectively. Additionally, they have a lower percentage of genomes with an O/E ratio below the 0.72 threshold (DnmA motif, 6.0%; mock DnmA motif, 2.6%; Fig. S4). Together, these data demonstrate that the DnmA motif is naive to the selective pressure observed with the BsuMM motif from an active RM system. Thus, if the DnmA-YeeB-YeeC gene cluster acts to restrict phage infection or amplification, the mechanism must be distinct from canonical RM systems such as BsuMM-BsuMR (27).

10.1128/mbio.03185-22.4 FIG S4 Motif enrichment in bacteriophage genomes. Genomes of bacteriophages from the Herelleviridae, Siphoviridae, Myoviridae, and Podoviridae families were analyzed for the presence of the DnmA motif (5′-GACGAG-3′), a mock DnmA motif (5′-CTGCTC-3′), and the active RM MTase BsuMM (5′-CTCGAG-3′). Each observed number of motifs was normalized to the expected number of motifs as determined by the compositional bias method (47). Points above or below the shaded region were considered over- or under-enriched (see Materials and Methods). The number of genomes with an O/E ratio below the threshold of 0.72 (*x* axis, “# below threshold”) is provided with percentages in parentheses. Each point represents an individual data point, boxplots represent the interquartile range of the data, and the density plots represent the distributions of the data values. Data plotted are for genomes containing at least 5 expected motifs; thus, *n* = 1,598 (CTGCTC), 1,702 (GACGAG), and 579 (CTCGAG). Download FIG S4, PDF file, 1.5 MB. Copyright © 2023 Fernandez et al. 2023 Fernandez et al. https://creativecommons.org/licenses/by/4.0/ This content is distributed under the terms of the Creative Commons Attribution 4.0 International license.

### The *dnmA-yeeB-yeeC* locus does not influence B. subtilis susceptibility to *Bacillus* phage Nf, *Bacillus* phage SBS-ΦJ, or bacillus virus Φ29.

**FIG 4:**
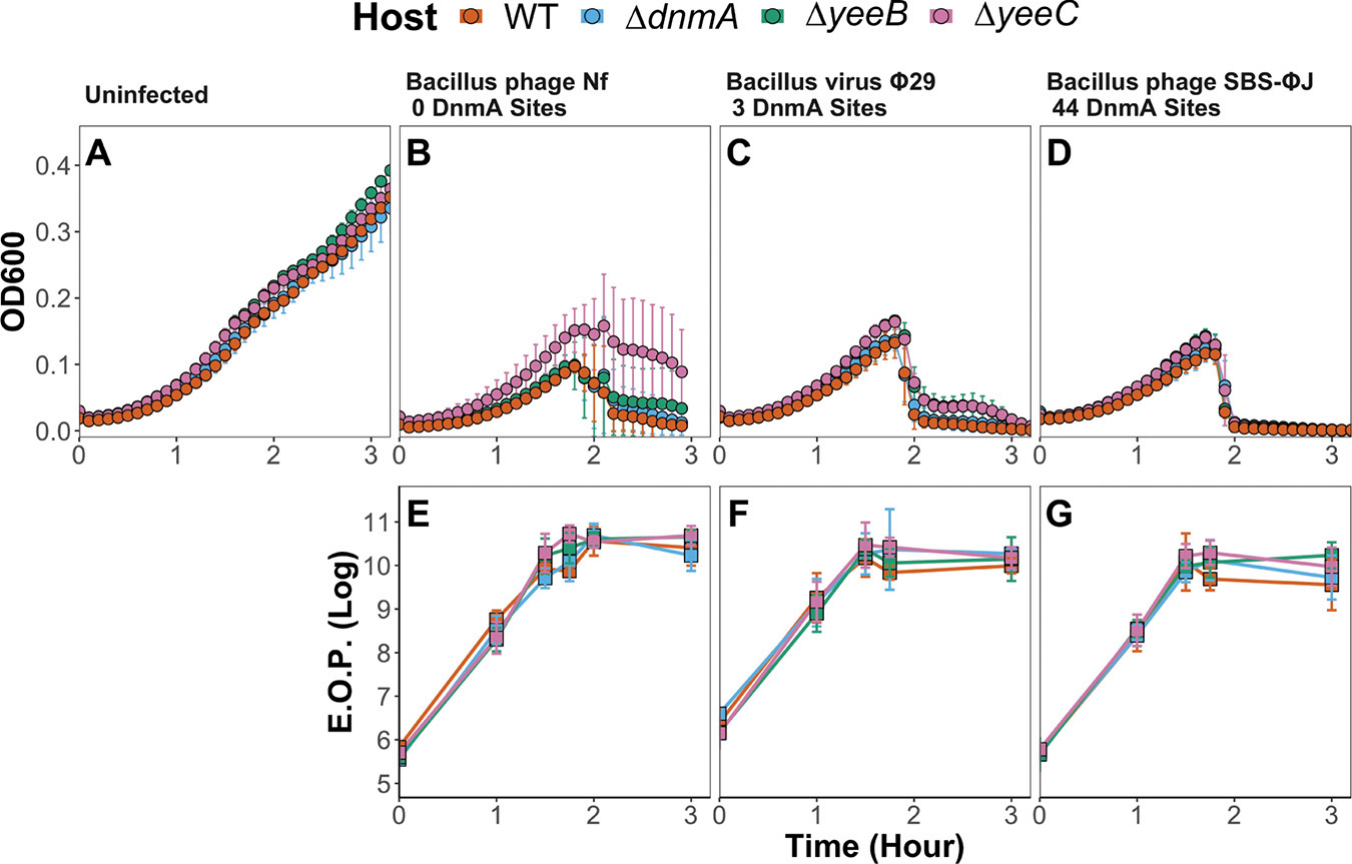
Neither the number of DnmA motifs nor the B. subtilis genotype influences predation by bacteriophage. (A to D) Growth curves of uninfected B. subtilis (A) and cells infected with: (B) *Bacillus* phage Nf, (C) *Bacillus* virus Φ29, and (D) *Bacillus* phage SBS-ΦJ. B. subtilis strains are differentiated by color. Cultures were pregrown, and phage addition (MOI of 0.1) occurred at time 0. Growth was monitored by OD_600_ measurements every 5 min for 3 h. Each point is the mean of 4 to 6 biological replicates, and error bars indicate standard deviation. (E to G) Separately, efficiency of plaquing (E.O.P.) was monitored over the same timescale after phage addition. Squares represent the mean E.O.P. value, and error bars denote the standard deviation.

Though the DnmA recognition site in bacteriophage genomes is not underenriched, the conservation of both gene arrangement and orientation suggests there is a selective advantage to maintaining *dnmA*, *yeeB*, and *yeeC*, such as limiting bacteriophage infection. We created single-gene deletions to directly test the hypothesis that lack of *yeeB* and *yeeC* will result in increased susceptibility to phage infection. Phage were chosen based on the enrichment and total number of DnmA sites within their respective genomes, including *Bacillus* phage Nf (0 sites), *Bacillus* virus Φ29 (3 sites, underenriched), and *Bacillus* phage SBS-ΦJ (44 sites, no enrichment). In the absence of phage, all strains grew similarly, indicating that single-gene deletions of *yeeB* and *yeeC* are not deleterious for growth (Fig. 4A). Regardless of strain, phage addition at *T*_0_ caused clearing of the culture within 2 h (Fig. 4B to D). Single-gene deletions did not alter phage production either, as the efficiency of plaquing (EOP) was similar between all strains and phages tested (Fig. 4E to G). Since *ΔyeeB* and *ΔyeeC* backgrounds had similar susceptibility to phage infection and EOP, these data indicate that the *dnmA*-*yeeB-yeeC* gene cluster is dispensable for protection against bacteriophage infection under the conditions tested here. Given that our results show (i) no evidence of underenrichment of the DnmA site in phage genomes; (ii) no difference in phage predation when comparing deletions of *dnmA*, *yeeB*, and *yeeC* to the WT; and (iii) no effect of *dnmA* on DNA uptake during natural transformation (13), we suggest that the *dnmA-yeeB-yeeC* cluster does not function as an RM or antiphage system. Instead, we suggest that DnmA is functionally an orphan MTase from a nonfunctional relic of an RM system.

## DISCUSSION

Genes encoding RM systems are found in many bacterial species, yet the functionality of most of these systems remains unknown (http://rebase.neb.com/rebase/) (8). In B. subtilis, the DNA methyltransferase DnmA was previously identified and characterized as an MTase that controls gene expression (13). Here, we explore how substrate composition and key amino acid residues in DnmA influences kinetics, and to expand the biological role of DnmA, we used single-molecule, bioinformatic, and genetic approaches to study DnmA function and regulation of dynamic movement and localization. Our *in vitro* and *in vivo* analyses of DnmA show that disrupting DNA binding by manipulating either DNA substrate availability or DNA binding residues influences DnmA-DNA interactions *in vitro* and DnmA localization *in vivo*. We show that DnmA is coincident with two genes with putative restriction functions; however, our data support the conclusion that DnmA does not participate as an antiphage system in B. subtilis.

We characterized the mobility and localization of DnmA *in vivo* through single-molecule tracking analyses, one of a handful of studies utilizing this technology to better understand prokaryotic DNA methylation *in vivo* (28). In unperturbed cells, DnmA is found throughout the center of the cell, likely interacting with the nucleoid, and has a positive correlation with the position of the replisome (Fig. 1). Negri and colleagues analyzed the mobility and localization of the DNA MTase M.Csp231I, which functions in an active RM system (28). Similar to our findings, M.Csp231I localizes throughout the nucleoid with a high probability of localizing near the mid- and quarter cell positions, suggesting a common DNA searching mechanism among DNA MTases in bacteria (28).

Single-molecule studies of DNA-binding enzymes in E. coli have suggested that the slower-moving enzyme molecules are involved in catalytic functions (29). However, due to the essential nature of the enzymes, catalytically inactive versions were not studied. Here, the use of the inactive DnmA variant DnmA[Y465A] allowed us to assess how catalysis influences DNA mobility and localization *in vivo*. Interestingly, DnmA[Y465A] and WT DnmA have similar percentages of slow-moving molecules, indicating that the slower-moving molecules are not necessarily enzymes involved in active catalysis (Fig. S1). Additionally, although we reasoned that disrupting DNA interactions in DnmA, either by amino acid substitution or manipulating available substrate pools, would result in a larger population of fast-moving molecules than in WT DnmA, we instead found that mobility remains largely unchanged compared to WT DnmA in unperturbed conditions except for upon HPUra treatment. Thus, our results highlight the importance of targeted amino acid substitutions and other approaches to better explain single-molecule results of catalytic enzymes.

Under high R-loop conditions (Δ*rnhC*), the localization pattern of DnmA switches from a concentration near the midcell and colocalization with the replisome to DnmA being negatively correlated with the replisome position (Fig. 1). Since DnmA binds RNA-DNA hybrids poorly *in vitro* (Fig. 2) and DNA binding is necessary for DNA methylation (15), our data suggest specific DNA interactions are necessary for proper DnmA localization and correlation with DnaX. This conclusion is supported by the even stronger negative DnaX correlation in DnmA[6AA*], which lacks the ability to recognize the DnmA recognition sequence *in vitro* (Fig. 2). While the mechanism for DnmA[6AA*] repositioning is not clear, protein sequestration and localization are used in bacteria to regulate enzymatic activity. In C. crescentus, the cell cycle regulating DNA MTase CcrM is inhibited by polar sequestration (30). While we do not observe strict polar DnmA localization under R-loop stress or in DnmA[6AA*], it is tempting to speculate that MTase repositioning in the cell represents a broad mechanism to negatively regulate DNA methylation and epigenetic gene expression in bacteria.

Morgan et al. found that B. subtilis DnmA (previously YeeA) is homologous to the type IIL MTase-REase protein MmeI, which has MTase and REase domains in a single polypeptide (31). The authors noted that DnmA did not encode a REase motif but was adjacent to YeeB and YeeC homologs. We expanded on this finding to include DnmA homologs from various species and found that genomes encoding DnmA likely encode two genes with helicase and nuclease functions (putative YeeB and YeeC homologs, respectively) within a 20-gene neighborhood, demonstrating that gene synteny and architecture are conserved (Fig. 3B). The putative recombinase genes *yefB* and *yefC* and the toxin-antitoxin pair *yeeF* and *yezG*, however, are not adjacent to DnmA at a high enough frequency for identification in our analysis. This result suggests that these genes represent B. subtilis-specific gene acquisitions. In S. pneumoniae, genes encoding the MTase specificity subunits, which direct the MTase to a given sequence, are subject to phase-variation through recombination, resulting in heterogenous methylation patterns in the genome (32). Thus, it is possible the adjacent recombinase genes may play a similar role in B. subtilis. In our previous characterization of DnmA, however, we observed homogenous methylation patterns under standard growth conditions (13). Additionally, we did not identify any sequence signatures suggestive of site-specific recombination flanking the low-GC region in the genome, such as inverted or direct repeats. The *yeeF* gene has an N-terminal LXG domain which allows for secretion through the type 7 secretion system (T7SS) encoded by the distally located genes *yukEDCB-yueBCD* (33). The C-terminal domain of YeeF encodes nuclease activity that is inactivated by the neighboring antitoxin YezG (34). Thus, our data suggest that this region represents a defunct mobile genetic element that is maintained through a selective benefit of DnmA and/or the antitoxin YezG.

The conservation and putative functions of *yeeB* and *yeeC* suggest a conserved function. We assessed the antiphage activity of DnmA, YeeB, and YeeC by testing whether single-deletion mutants had any effect on host survival and/or bacteriophage amplification. Despite using bacteriophages with a range of DnmA motifs in their genome, the single-deletion mutants had no effect on bacteriophage-mediated host killing or production (Fig. 4), leading us to conclude that DnmA is part of a remnant of a nonfunctional RM system. This observation is important because of the pervasive occurrence of MTases and DNA methylation in the domain *Bacteria* (8).

DnmA-YeeB-YeeC homologs in the marine microorganism Vibrio crassostreae were identified in a recent study (35). Deletion mutations of *dnmA* and *yeeB* caused an increase in bacteriophage sensitivity to some subclades of bacteriophage, while having no effect when other subclades were used (35). An amino acid alignment of DnmA, YeeB, and YeeC from B. subtilis and *V. crassostreae* shows that all three proteins share high sequence homology in putative active site domains (Fig. S5). However, YeeB and YeeC from B. subtilis are missing several-amino acid-long stretches in the C-terminal domain. Therefore, it is possible that YeeB and YeeC in B. subtilis are missing critical residues necessary for antiphage function. Our data suggest that numerous bacterial MTases detected in the bacterial methylome also originate from defunct phage defense systems, similar to *dnmA* in B. subtilis (8). These defunct defense systems could have maintained an active MTase either for epigenetic control or due to the presence of a toxin-antitoxin system that selects for the acquired region while losing restriction activity.

10.1128/mbio.03185-22.5 FIG S5 Alignment of DnmA, YeeB, and YeeC homologs from Vibrio crassostreae and Bacillus subtilis. Identical residues for each alignment are in blue. Amino acids missing in B. subtilis homologs are highlighted with red boxes. Motifs involved in protein function are highlighted with black boxes with the motif name printed above the box. (A) Alignment of DnmA amino acid sequences from 298 to 515 for *V. crassostreae* and 289 to 507 for B. subtilis. Boxes highlight the residues found in the two conserved active sites FGG and NPPY. DnmA*_Vcr_* is 57% similar to DnmA*_Bsu_*. (B) Alignment of amino acid sequences of YeeB homologs. Boxes highlight conserved motifs found in domain 1 and domain 2, respectively, of superfamily 2 (SF2) helicases. YeeB*_Vcr_* is 56.76% similar to YeeB*_Bsu_*. (C) Alignment of amino acid sequences of YeeC homologs. The black box highlights conserved residues found in GIY-YIG nuclease family protein/T5orf172-domain containing proteins. YeeC*_Vcr_* is 32.96% similar to YeeC*_Bsu_*. Download FIG S5, PDF file, 1.4 MB. Copyright © 2023 Fernandez et al. 2023 Fernandez et al. https://creativecommons.org/licenses/by/4.0/ This content is distributed under the terms of the Creative Commons Attribution 4.0 International license.

## MATERIALS AND METHODS

### Cloning and strain construction.

The expression vector for WT DnmA (pTN002) was constructed as previously described (13).

**(i) pNF025.** The protein expression vector harboring Y465A DnmA was constructed by amplifying the pE-SUMO backbone using oligos oLM1 and oLM2. The gene encoding the Y465A DnmA variant was assembled from two fragments: fragment 1 (oTMN005-oNLF079) and fragment 2 (oNLF080-oTMN007). The mutation causing the Y465A substitution was incorporated into the primers oNLF079 and oNLF080. The two fragments were assembled using splice by overlap extension (SOE) PCR, and the resulting assembled fragment was gel extracted, mixed with pE-SUMO in a 3:1 insert/vector molar ratio, assembled by Gibson assembly, and used to transform E. coli TOP10 cells. Transformants were screened by colony PCR, and positive clones were purified, sequenced by whole-plasmid sequencing (Plasmidsaurus), and used to transform BL21(DE3) cells.

**(ii) pNF024.** The protein expression vector harboring the DNA binding variant (M620A, N748A, K470A, L780A, K781A, D783A) was constructed by first synthesizing a DNA fragment with all mutations for the six amino acid substitutions (Twist Biosciences). The gene encoding the DNA binding DnmA variant was assembled from two fragments: fragment 1 (oTMN005-oNLF265) and fragment 2 (oNLF264-oTMN007). The fragments were assembled into pE-SUMO in the same fashion as pNF025.

**(iii) pNF003.** A CRISPR/CAS9 deletion vector targeting the *erm* cassette (pLVG03) with WT DnmA-PAmCherry replacement (protocol adapted from reference 36). The CRISPR backbone was amplified from pLVG012 using oPEB232-oPEB234. The replicon and antibiotic selection markers were amplified from pPB41 using oPEB217-oPEB218. DnmA-PAmCherry was generated by fusing upstream of DnmA to the last residue before the stop codon (oNLF029-oLVG029B, WT genomic DNA (gDNA) template), linker plus PAmCherry (oLVG028A-oLVG028B, pLVG012 template), and downstream of DnmA (oLVG029C-oNLF032 WT gDNA template) by SOE PCR. The amplicon of the correct size of the fused fragments was gel extracted and mixed with the oPEB232-oPEB234 and oPEB217-oPEB218 fragments in a 1:1:1 ratio and assembled using Gibson Assembly mastermix (NEB) for 1 h at 50^ο^C and then heat-shocked into MC1061 E. coli cells. Transformants were selected for on LB supplemented with spectinomycin (100 μg/mL) and screened for correct assembly by colony PCR using oligonucleotides oPEB227 and oNLF236 and sequenced (Plasmidsaurus).

**(iv) pNF023.** A CRISPR/CAS9 deletion vector targeted the *erm* cassette (pLVG03) with DnmA DNA binding variant-PAmCherry replacement. The CRISPR backbone was amplified in the same manner as pNF003. The DNA fragment with all mutations for the six amino acid substitutions was synthesized (Twist Biosciences) and amplified with oNLF264-oNLF267. The DnmA binding variant fused to PAmCherry was assembled from four fragments. oNLF029-oNLF265 was used to amplify 1 kb upstream of the start codon of DnmA to upstream of the mutated region using wild-type genomic DNA as the template (fragment 1). oNLF266-oLVG029B was used to amplify the region downstream of the mutant region up to the stop codon (fragment 2). oLVG028A-oLVG28B was used to amplify the linker region and PAmCherry (fragment 3). oLVG029C-oNLF032 was used to amplify 1 kb downstream of the *dnmA* stop codon using wild-type genomic DNA as the template (fragment 4). The four DnmA fragments were gel extracted and fused together by SOE PCR. The amplicon of the correct size of the fused fragments was gel extracted and mixed with the oPEB232-oPEB234 and oPEB217-oPEB218 fragments in a 1:1:1 ratio and assembled using Gibson Assembly mastermix (NEB) for 1 h at 50^ο^C and then heat-shocked into MC1061 E. coli cells. Transformants were screened for correct assembly by colony PCR and sequenced (Plasmidsaurus).

**(v) pNF026.** A CRISPR/CAS9 deletion vector targeted the *erm* cassette (pLVG03) with Y465A DnmA-PAmCherry replacement. The CRISPR backbones were generated in the same manner as pNF003. The Y465A DnmA variant fused to PAmCherry was assembled from four fragments. oNLF029-oNLF079 was used to amplify 1 kb upstream of the start codon of DnmA to residue 465 using wild-type genomic DNA as the template (fragment 1). oNLF080-oLVG029B was used to amplify the region downstream of the residue 465 region up to the stop codon (fragment 2). The mutation causing the Y465A substitution was incorporated into the primers oNLF079 and oNLF080. oLVG028A-oLVG28B was used to amplify the linker region and PAmCherry (fragment 3). oLVG029C-oNLF032 was used to amplify 1 kb downstream of the *dnmA* stop codon using wild-type genomic DNA as the template (fragment 4). The four DnmA fragments were gel extracted and fused together by SOE PCR. The amplicon of the correct size of the fused fragments was gel extracted and mixed with the oPEB232-oPEB234 and oPEB217-oPEB218 fragments in a 1:1:1 ratio and assembled using Gibson Assembly mastermix (NEB) for 1 h at 50^ο^C and then heat-shocked into MC1061 E. coli cells. Transformants were screened for correct assembly by colony PCR and sequenced (Plasmidsaurus).

**(vi) Bacillus subtilis strains.** B. subtilis Δ*yeeB* and Δ*yeeC* were constructed in the PY79 background by natural transformation using purified genomic DNA from strains BKE06770 and BKE06780, respectively, courtesy of the Bacillus Genetic Stock Center (http://bgsc.org, (37)) and selecting for transformants on LB plates supplemented with erythromycin. The erythromycin cassettes in the resulting transformants were removed by transformation of the plasmid pDR244, which harbors a site-specific recombinase that catalyzes recombination between the lox-sites flanking the erythromycin cassette. The DnmA-PAmCherry strain was made via CRISPR-CAS genome editing (36). Transformants were then cured of the CRISPR/CAS9 deletion vectors by incubation at 42°C overnight. Strains that were spectinomycin and erythromycin sensitive were stored and used for experiments. The strains, plasmids, and oligonucleotides used in this study can be found in Tables S1, 2, and 3, respectively.

10.1128/mbio.03185-22.6 TABLE S1 All strains used in this study (each strain is a derivative of B. subtilis WT strain PY79). Download Table S1, DOCX file, 0.02 MB. Copyright © 2023 Fernandez et al. 2023 Fernandez et al. https://creativecommons.org/licenses/by/4.0/ This content is distributed under the terms of the Creative Commons Attribution 4.0 International license.

### DnmA purification.

An overnight culture of pTMN14 was started by inoculating 5 mL of LB supplemented with kanamycin at a 10-μg/mL final concentration and incubating the mixture at 37°C while shaking at 220 rpm. The next day, the overnight culture was diluted 1:500 in 500 mL LB supplemented with kanamycin and grown at 37°C while shaking at 220 rpm for 2.5 h. Then, 1 mM IPTG (isopropyl-β-d-thiogalactopyranoside; final concentration) was added, and protein production was induced for 3 h at 37°C and 220 rpm. After 3 h, the culture was pelleted, snap-frozen in a dry ice/ethanol bath, and stored at −80°C overnight. Pellets were then thawed on ice and resuspended in 20 mL lysis buffer (50 mM Tris, pH 8, 300 mM NaCl, 10% sucrose, 10 mM imidazole) and lysed by sonication (Branson SFX250 sonifier, 70% amplitude, 30 cycles of 10 s on and 10 s off on ice.). The lysed cell solution was clarified by centrifugation (45 min, 15,000 × *g*, 4°C). During clarification, 10 mL Ni-NTA resin (Qiagen) was equilibrated and washed with deionized (DI) water followed by 2 column volumes of wash buffer (20 mM Tris, pH 8, 10% glycerol, 20 mM imidazole, 2M NaCl) at room temperature. Clarified lysate was loaded onto the Ni-NTA column and was allowed to flow through by gravity, followed by washing with 6 column volumes wash buffer. After the last wash, 10 mL elution buffer (50 mM Tris, pH 8, 150 mM NaCl, 400 mM imidazole) was added to the column and collected. The protein solution was buffer exchanged by dialysis in dialysis buffer (50 mM Tris, pH 8, 150 mM NaCl, 5% glycerol) overnight at 4°C. The protein solution was then treated with small ubiquitin-like modifier (SUMO) protease by adding purified SUMO protease and 1 mM dithiothreitol (DTT; final concentration) and incubating the mixture at room temperature for 2 h. The protein solution was buffer exchanged into dialysis buffer to remove excess DTT, and the SUMO tag and protease were removed from the solution by applying the purified protein solution to a 10-mL Ni-NTA column and collecting the flowthrough. Purified and tagless protein was buffer exchanged into dialysis buffer (without glycerol) and concentrated using Amicon filters (10-kDa cutoff). Glycerol (25% final concentration) was added to the purified, concentrated, and tagless protein solution and stored as 25-μL aliquots at 80°C.

### Electrophoretic mobility shift assay (EMSA).

Production of m6A in oNLF001 was carried out by Integrated DNA Technologies (IDT), and it was determined to be 98% pure by electrospray ionization mass spectrometry (IDT). For annealing, solutions of the unmethylated probe (oligonucleotides oTMN67/oTMN68), methylated probe (oNLF001/oTMN68), and the RNA-DNA hybrid (oTMN67/oJRR271) were mixed at a final concentration of 50 nM and incubated at room temperature overnight, covered from light. Purified ScoC was mixed in a binding reaction consisting of 5× EMSA reaction buffer (500 mM Tris-HCl, pH 8, 1.25 M NaCl, 10% glycerol [vol/vol]) and 5 nM (final concentration) annealed oligonucleotides. Reaction mixtures were incubated for 30 min at 25°C. Afterward, 8 μL of the mixture was loaded onto and resolved via prerun 6% native-PAGE, which was performed covered from light and on ice for 60 min at 100 V in 1× Tris/Borate/Ethylenediaminetetraacetic acid (EDTA). The samples were visualized with the LI-COR Odyssey imager. Intensities of the shifted and unshifted bands were quantified using Fiji image software using the gel feature (38). The fraction bound was calculated by first subtracting the background signal (region of gel with no band) from the intensity measurement of each band. The intensity of the bound substrate was divided by the sum of intensities of the bound and unbound substrate, yielding the fraction bound. Fraction bound data were modeled using the four-parameter log-logistic function in the drc package for R. and the effective concentration for half maximal binding (EC_50_) was measured for each replicate (39).

### Flow cytometry.

Strains of interest were struck out on LB agar plates and incubated 16 h overnight at 30°C. The next day, 6 isolated colonies were inoculated in 250 μL LB in wells of a 96-well plate and grown at 37°C in an orbital shaker at 250 rpm until the early exponential phase. Cultures were then moved to microcentrifuge tubes and diluted 1:1 with 200 μL sterile 1× phosphate-buffered saline (PBS), and single-cell fluorescence was measured using an Attune NxT acoustic focusing cytometer (Thermo Fisher Scientific). Fluorescence data were acquired from 200,000 cells with the following settings: flow rate, 25 μL/min; forward scatter (FSC) voltage, 200; side scatter (SSC) voltage, 250; blue light detector 1 (BL1) voltage, 250.

### Live-cell single-molecule imaging.

B. subtilis strains expressing DnmA-PAmCherry (PY79 and Δ*rnhC* PY79) and DnmA variants (DnmA[Y465A]-PAmCherry and DnmA-6AA*-PAmCherry) were grown overnight on LB agar plates at 37°C. The cells were washed from the plate with filtered S7_50_ minimal medium and inoculated in filtered S7_50_ minimal medium at an optical density at 600 nm (OD_600_) of ~0.1, followed by growth with shaking at 200 rpm at 30°C for ~4 h until reaching an OD_600_ of ~0.5 to 0.6 (S7_50_ minimal medium: 1× S7_50_ salts [10× S750 salts: 0.5 M MOPS [morpholinepropanesulfonic acid], pH 7.4, 100 mM ammonium sulfate, 50 mM potassium phosphate monobasic, filter sterilized]), 1× metals [100× metals: 0.2 M MgCl_2_, 70 mM CaCl_2_, 5 mM MnCl_2_, 0.1 mM ZnCl_2_, 100 μg/mL thiamine HCl, 2 mM HCl, 0.5 mM FeCl_3_ (added last to prevent precipitation), (filter sterilized)], 1% glucose, 0.1% glutamate, 40 μg/mL tryptophan, 40 μg/mL phenylalanine. Experiments in 6-(p-hydroxyphenylazo)-uracil (HPUra) were done by adding HPUra at a final concentration of 162 μM to the culture immediately before imaging. Coverslips were cleaned via argon plasma etching (PE-50, plasma etch) for 30 min, and 2% agarose pads were prepared with freshly made, filtered S7_50_ medium to reduce background fluorescent signals. Cells were pipetted onto agarose pads and sandwiched between coverslips for imaging. Once prepared, the sample was mounted on a wide-field inverted microscope (Olympus IX71, Melville, NY) for single-molecule imaging.

Prior to imaging, the cells and background were photobleached with a 561-nm laser (Sapphire 561-50, Coherent, Bloomfield, CT) for 2 min at a power density of 630 W/cm^2^. Single DnmA-PAmCherry molecules were photoactivated with 400-ms pulses of a 405-nm laser (Cube 405-100, Coherent) with a power density of 21.6 W/cm^2^ at the start of the imaging and after photobleaching. Photoactivated DnmA-PAmCherry molecules were imaged with a 561-nm laser with a power density of 69.2 W/cm^2^ and appropriate dichroic and long-pass filters. Fluorescence was collected via a 1.40 NA 100× oil-immersion phase-contrast objective and detected with a 512 by 512-pixel electron multiplying charge-coupled device camera (Photometrics, Acton, MA). Images were recorded with 40-ms exposure time.

### Single-molecule detection, tracking, and analysis.

Phase-contrast images were used to provide a reference mask for single-molecule detection and fitting within cell boundaries. Single-molecule fitting was done via the single-molecule localization by local background subtraction (SMALL-LABS) algorithm (16). The fit positions were connected into trajectories using the Hungarian algorithm (17).

The diffusion coefficients for each trajectory were fitted through (40) MSD = 4*Dτ* + 2*σ*^2^, where MSD is the squared displacement, *τ* is the time lag, and *σ* is the localization precision. The normalized heatmaps in Fig. 1E to J include the positions of all single molecules in all cells under each condition. First, the cell outlines were determined from segmentation of the phase contrast images, and then the Feret properties of each cell were calculated (MATLAB function bwferet) to determine the long and short axis of each cell. The single-molecule localizations of DnmA in each cell were projected onto the corresponding cell’s long and short axes to acquire the relative position of that molecule in the cell. Based on assuming the cells are symmetrical along the long and short axes, the 2D relative position of each single-molecule location was symmetrized along the two axes.

The curve fitting for the histogram of diffusion coefficients in Fig. 1D and Fig. S2A depicts the single-component Gaussian fitting, and the logarithm of single-trajectory diffusion coefficients are weighted based on track length. Figure S2B to F depict the 2-component Gaussian fitting of the logarithm of single-trajectory diffusion coefficients in Fig. S2A. The Spot-On algorithm was applied to fit the probability density function of single-molecule displacements to a 2-state model and a 3-state model to get the weight fraction of each component for WT DnmA (41). For Spot-On analysis of the other data sets, the fitted diffusion coefficient range for each state is fixed within the confidence interval of the corresponding state’s WT DnmA diffusion coefficient value to enable a direct comparison of the weight fraction of each state between different data sets.

### Percent GC content.

The percent GC content of the B. subtilis PY79 genome between 0.68 and 0.725 mega-base pairs (mbps) was calculated by generating a sliding window bed file using the BEDOPS subcommands –chop (1,000 bp window size) and –stagger (10 bp step size) (42). The BEDtools suite subcommand nuc was used to extract the nucleotide content (including percent GC) from the sliding window bed file (43).

### Gene neighborhood analysis.

The amino acid sequence of B. subtilis DnmA was used as a BLAST query using the Enzyme Function Initiative Genome Neighborhood Tool (GNT) (44). The GNT output provides neighborhood diagrams consisting of 10 genes upstream and 10 genes downstream of the target gene for each *dnmA* homolog. A total of 368 neighborhoods and associated data, including positions of genes and protein family (PFAM) identifies, were downloaded for further analysis. The number of defense-associated PFAM IDs within the neighborhood were calculated by comparing the neighborhood PFAM IDs to a list of PFAM IDs associated with antiphage defense systems (Table S4, adapted from reference 45). The expected number of defense-associated PFAMs per neighborhood was calculated by repeating this analysis using 20 random gene neighborhoods in a sample of 50 genomes from the 368 genomes identified in the initial BLAST search. The positions and identities of the top five most frequent *dnmA* homolog neighbors were counted using the EFI-GNT data (Table S4).

10.1128/mbio.03185-22.7 TABLE S2 Description and construction of all plasmids used in this study. Download Table S2, DOCX file, 0.01 MB. Copyright © 2023 Fernandez et al. 2023 Fernandez et al. https://creativecommons.org/licenses/by/4.0/ This content is distributed under the terms of the Creative Commons Attribution 4.0 International license.

10.1128/mbio.03185-22.8 TABLE S3 All oligonucleotides used in this study for cloning or EMSA analysis (RNA is noted with an asterisk [*], and 5′ infrared dye is denoted as 5IRD800 or 5IRD700 with excitation at 800 nm or 700 nm, respectively). Download Table S3, DOCX file, 0.01 MB. Copyright © 2023 Fernandez et al. 2023 Fernandez et al. https://creativecommons.org/licenses/by/4.0/ This content is distributed under the terms of the Creative Commons Attribution 4.0 International license.

10.1128/mbio.03185-22.9 TABLE S4 The defense-associated Pfam (xlsx file), the Genome Neighborhood Tool output (xlsx file), and phage names (xlsx file) (all of these items were used to generate Fig. 3). Download Table S4, XLSX file, 1.1 MB. Copyright © 2023 Fernandez et al. 2023 Fernandez et al. https://creativecommons.org/licenses/by/4.0/ This content is distributed under the terms of the Creative Commons Attribution 4.0 International license.

### Analysis of MTase sites in bacteriophages.

Complete genomic sequences of 1,913 bacteriophages from 4 families (Herelleviridae, Siphoviridae, Myoviridae, and Podoviridae) were downloaded from the NCBI website (Table S4). Expected counts of the MTase motifs BsuMM (5′-CTCGAG-3′) and DnmA (5′-GACGAG-3′) and a mock DnmA motif (5′-CTGCTC-3′) were calculated using the compositional bias method, and an observed/expected ratio was calculated by dividing the observed number of motifs by the expected number of motifs (26, 46, 47). Genomes with fewer than 5 expected motifs were due to the aberrant effects of low expected values on the O/E ratio. An O/E threshold of <0.72 and >1.30 was used to determine genomes which have under- or overenriched MTase sites (26, 46). The threshold was chosen to include 95% of the datapoints for the control motif 5′-CTGCTC-3′.

### Phage propagation.

Phage solutions from the Bacillus subtilis Stock Center (*Bacillus* phage Nf, BGSCID 1P19, *Bacillus* phage SBS-ΦJ BGSCID 1P47, and *Bacillus* phage Φ29 BGSCID 1P45) were diluted and spotted on LB plates confluent with WT B. subtilis PY79 and incubated overnight at 30°C. The next day, WT B. subtilis was grown in LB at 30°C while shaking at 220 rpm until turbid. An isolated plaque was picked from the plate, added to the culture, and grown until the culture lysed. The lysate was collected and centrifuged, the lysate was mixed 1:1 with chloroform, and the aqueous phase was collected and stored at 4°C. To quantify phage titers, phage solutions were serially diluted in LB, and 20 μL was spotted on LB plates using WT B. subtilis as the host. PFU were counted after overnight incubation at 25°C to calculate the titers for each phage.

### Phage infection.

WT and mutant B. subtilis strains were struck out onto LB plates in triplicate from frozen stocks and incubated overnight (~18 h) at 30°C. The next day, cells were collected by washing the plate with LB. The resulting cell suspension was adjusted to a starting OD_600_ of 0.050 in 1.5 mL LB in microcentrifuge tubes. Then,100 μL of culture was delivered to an individual well of a clear, sterile 96-well plate (Thermo Scientific). Two technical replicates were included for each biological replicate. The plates were then incubated at 37°C with shaking at 240 rpm until the OD_600_ reached approximately 0.150, which corresponds to ~1 × 10^7^ CFU/mL. At this point, phage (multiplicity of infection [MOI], 0.1) or LB (MOI,= 0) was added to the appropriate wells, and growth was measured using a Tecan Infinite M200 plate reader by monitoring the OD_600_ every 5 min for 3 h. The plates were mixed in between reads at 140 rpm using the linear mode. Data points represent the average OD_600_, and error bars represent the standard deviation for each strain.

### Plaque assays.

WT and mutant B. subtilis strains were prepared as described above and used to inoculate 2 mL LB at a starting OD_600_ of 0.050 in polystyrene culture tubes (Fischer, 17 mm by 100 mm). Cultures were allowed to grow at 37°C and were aerated by shaking at 220 rpm until the OD_600_ reached ~0.150 or ~1 × 10^7^ CFU/mL. Phage were added at an MOI of 0.1, and phage titers were measured at the time of addition and after 3 h of growth at 37°C. Phage samples were diluted in corresponding B. subtilis culture (OD_600_, 0.7), and 20 μL was spotted on LB plates. The PFU per mL were calculated for time points between 0 and 3 h.

### Data availability.

All code for analyzing heterogeneous diffusion and generated normalized heatmaps is publicly available at https://github.com/BiteenMatlab/SingleMoleculeDataAnalysis (48). All code for analyzing genome neighborhood analysis is publicly available at https://github.com/n-fernandez-1/DnmA_Manuscript_Scripts (49). The raw data used to generate Fig. 2 and Fig. S2 are publicly available at http://dx.doi.org/10.5281/zenodo.6014353.
