# Supplementary figures and images for: DNA Methylation and RNA-DNA Hybrids Regulate the Single-Molecule Localization of a DNA Methyltransferase on the Bacterial Nucleoid
Source: mBio. 2023 Jan 16;14(1):e03185-22. doi: 10.1128/mbio.03185-22 (PMC9973331; doi:10.1128/mbio.03185-22)

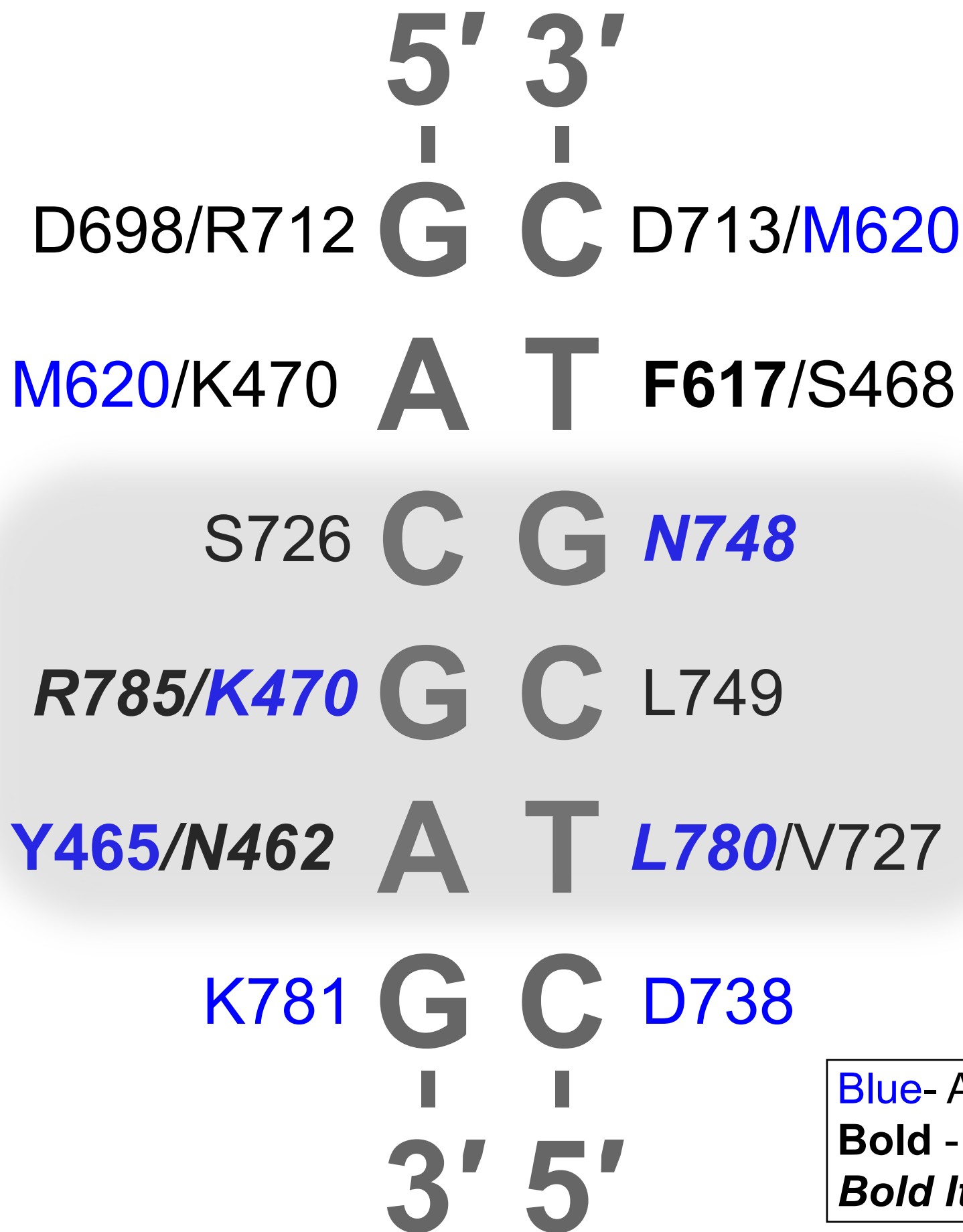

Blue- Alanine Substitution  
 Bold - Similar Residue  
 Bold *Italic* - Identical Residue

Supplement: FIG S3 [file mbio.03185-22-s0003.pdf]

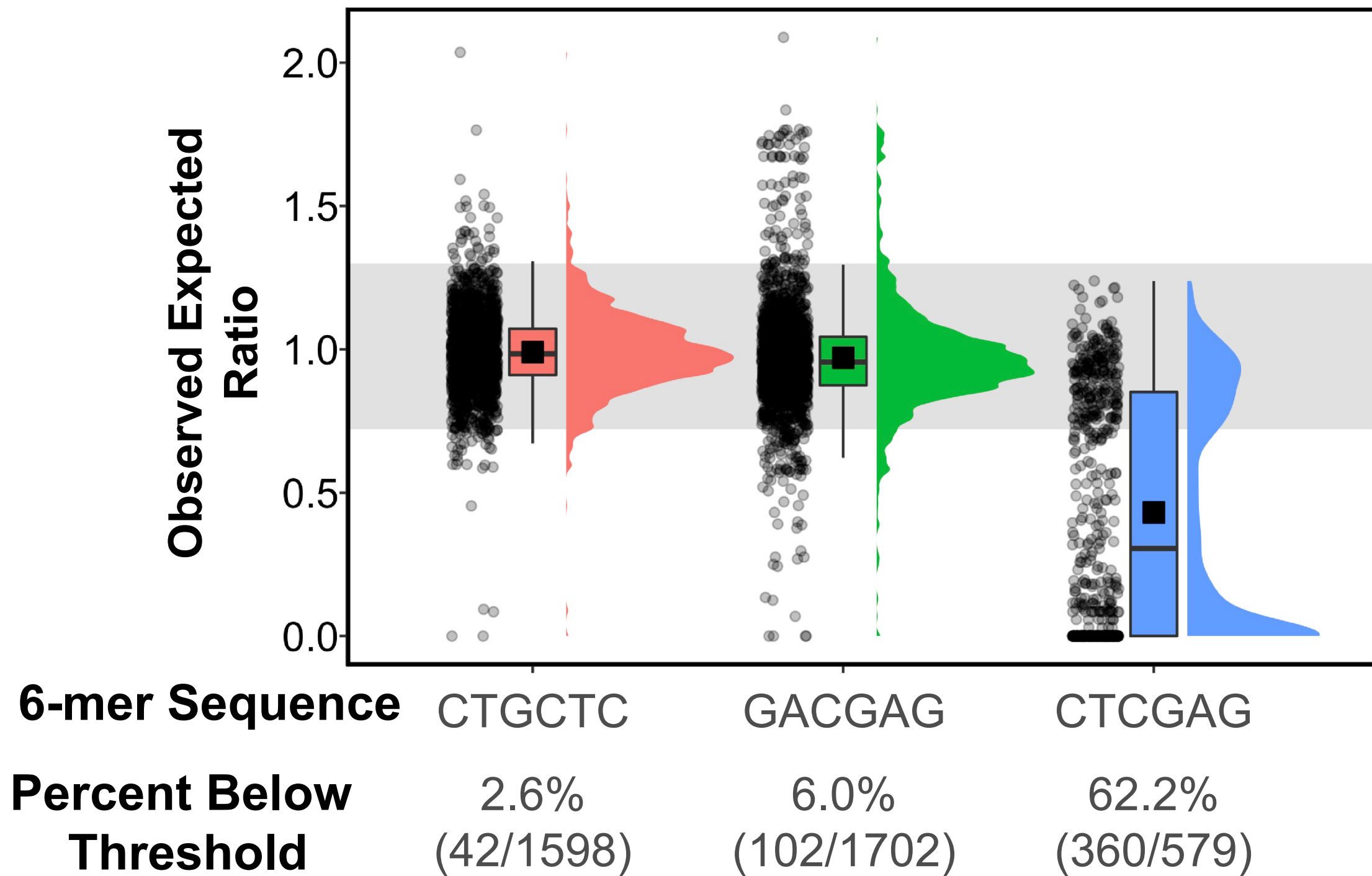

Supplement: FIG S4 [file mbio.03185-22-s0004.pdf]
