# Supplementary material for: DNA Methylation and RNA-DNA Hybrids Regulate the Single-Molecule Localization of a DNA Methyltransferase on the Bacterial Nucleoid
Source: mBio. 2023 Jan 16;14(1):e03185-22. doi: 10.1128/mbio.03185-22 (PMC9973331; doi:10.1128/mbio.03185-22)
Supplement: FIG S5 [file mbio.03185-22-s0005.pdf]

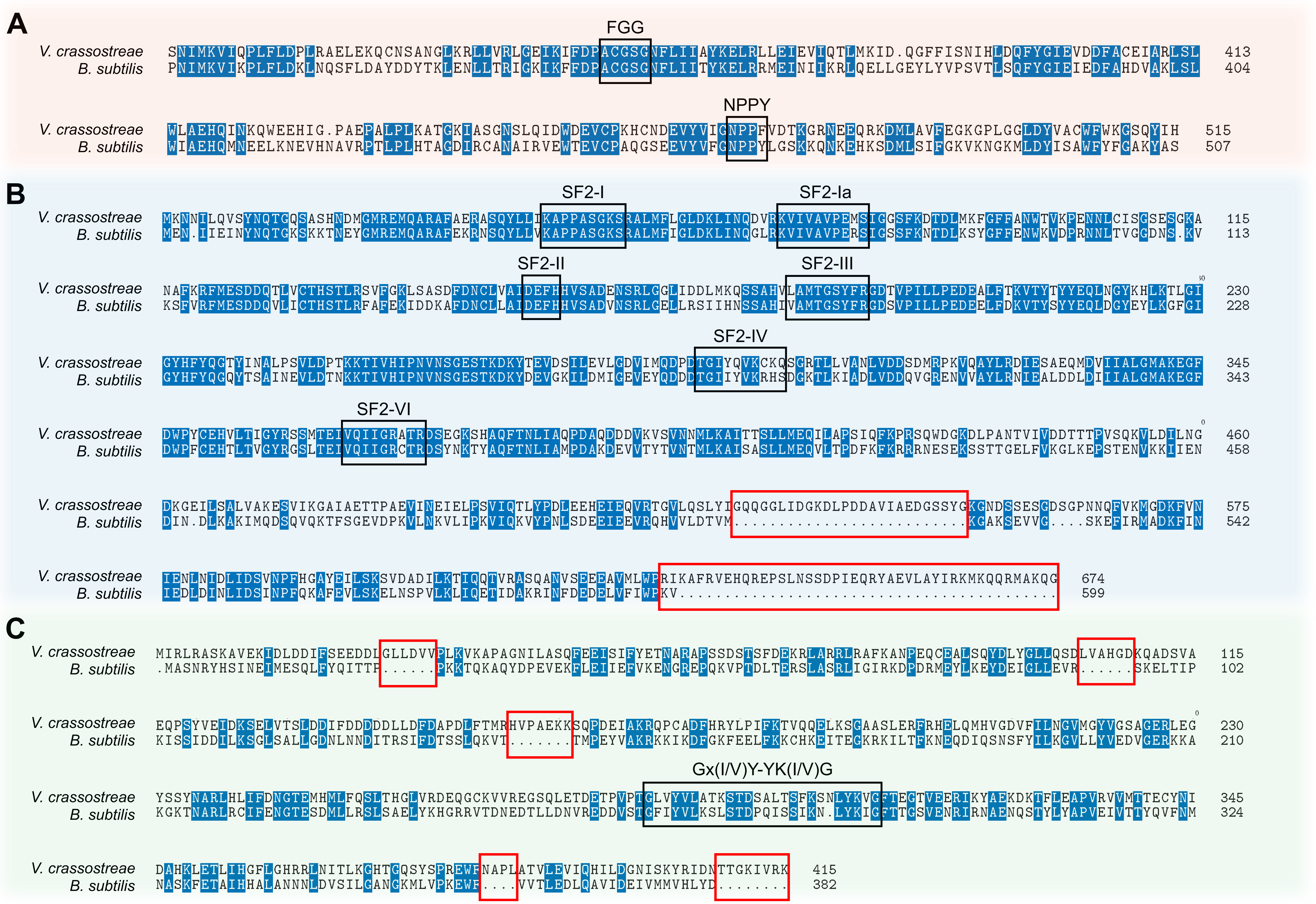

A
FGG
V. crassostreae 413
B. subtilis 404
NPPY
V. crassostreae 515
B. subtilis 507
B
SF2-I
SF2-Ia
V. crassostreae 115
B. subtilis 113
SF2-II
SF2-III
V. crassostreae 230
B. subtilis 228
SF2-IV
V. crassostreae 345
B. subtilis 343
SF2-VI
V. crassostreae 460
B. subtilis 458
V. crassostreae 575
B. subtilis 542
V. crassostreae 674
B. subtilis 599
C
V. crassostreae 115
B. subtilis 102
V. crassostreae 230
B. subtilis 210
Gx(I/V)Y-YK(I/V)G
V. crassostreae 345
B. subtilis 324
V. crassostreae 415
B. subtilis 382
