# Supplementary material for: DNA Methylation and RNA-DNA Hybrids Regulate the Single-Molecule Localization of a DNA Methyltransferase on the Bacterial Nucleoid
Source: mBio. 2023 Jan 16;14(1):e03185-22. doi: 10.1128/mbio.03185-22 (PMC9973331; doi:10.1128/mbio.03185-22)
Supplement: TABLE S1 [file mbio.03185-22-s0006.docx]

## Supplemental Table S1: Strains used in this study.

| **Strain** | **Genotype/description** | **Source** |
| --- | --- | --- |
| JWS10 | PY79 Wild type | (51) |
| TMN5 | *∆dnmA* | (13) |
| NF011 | *∆yeeB* | This Study |
| NF012 | ∆*yeeC* | This Study |
| NF037 | *dnmA::dnmA-PAmCherry* | This Study |
| NF035 | dnmA::Y465A DnmA-PAmCherry | This Study |
| NF036 | dnmA::DNA binding mutant DnmA-PAmCherry | This Study |
| TMN131 | *dnmA::dnmA-PAmCherry; ∆rnhC* | (51) |
| NF038 | NF035 *amyE::scpA-gfp* | This Study |
| NF039 | NF036 *amyE::scpA-gfp* | This Study |
| NF040 | NF037 *amyE::scpA-gfp* | This Study |
| NF042 | NF037 *amyE::pxyl-dnaX-mCitrine* | This Study |
| NF045 | NF035 *amyE::pxyl-dnaX-mCitrine* | This Study |
| NF046 | NF036 *amyE::pxyl-dnaX-mCitrine* | This Study |
| NF061 | NF037 *∆rnhC* | This Study |
| NF063 | NF061 *amyE::pxyl-dnaX-mCitrine* | This Study |
