# Supplementary material for: DNA Methylation and RNA-DNA Hybrids Regulate the Single-Molecule Localization of a DNA Methyltransferase on the Bacterial Nucleoid
Source: mBio. 2023 Jan 16;14(1):e03185-22. doi: 10.1128/mbio.03185-22 (PMC9973331; doi:10.1128/mbio.03185-22)
Supplement: TABLE S2 [file mbio.03185-22-s0007.docx]

## Supplemental Table S2: Plasmids used in this study.

| **Plasmid** | **Selection** | **Description** | **Source** |
| --- | --- | --- | --- |
| pPB41 | Amp;Spec | CRISPR-Cas vector backbone | (41) |
| pDR244 | Spec | Temperature sensitive vector harboring Cre recombinase | (40) |
| pLVG02 | Amp;Spec | pPB41 with *erm* cassette targeting protospacer. | This study |
| pLVG03 | Amp;Spec | pLVG02 with DnmA-PAmCherry editing template | This study |
| pNF026 | Amp;Spec | CRISPR KO construct targeting the erm cassette with Y465A DnmA-PAmCherry replacement | This study |
| pNF023 | Amp; Spec | CRISPR KO construct targeting the erm cassette with DnmA DNA binding mutant-PAmCherry replacement. | This study |
| pNF024 | Kan | Protein expression vector harboring DNA binding mutant DnmA. | This study |
| pNF025 | Kan | Protein expression vector harboring Y465A DnmA. | This study |
| pNF003 | Amp; Spec | CRISPR KO construct targeting the erm cassette with WT DnmA-PAmCherry replacement | This study |
