# Supplementary material for: DNA Methylation and RNA-DNA Hybrids Regulate the Single-Molecule Localization of a DNA Methyltransferase on the Bacterial Nucleoid
Source: mBio. 2023 Jan 16;14(1):e03185-22. doi: 10.1128/mbio.03185-22 (PMC9973331; doi:10.1128/mbio.03185-22)
Supplement: TABLE S3 [file mbio.03185-22-s0008.docx]

## Supplemental Table S3: Oligonucleotides used in this study.

| **Oligo Name** | **Sequence (5’-3’)** | **Use** |
| --- | --- | --- |
| oPEB217 | GAACCTCATTACGAATTCAGCATGC | Strain Construction |
| oPEB218 | GAATGGCGATTTTCGTTCGTGAATAC |  |
| oPEB232 | GCTGTAGGCATAGGCTTGGTTATG |  |
| oPEB234 | GTATTCACGAACGAAAATCGCCATTCCTAGCAGCACGCCATAGTGACTG |  |
| oLVG028A | GCGGGTTCTGGAGGTGAAGCTGAACTGTTTCTAAAGGCGAAGAAG |  |
| oLVG028B | TTTGTAAAGTTCATCCATGCCGCC |  |
| oLVG029A | CCAGGGCTGCAGGAATTCGACTCTCCATTGACCTCCTGCAATC |  |
| oLVG029B | CAGCTTCACCTCCAGAACCCGCCCGTTCTGTCATTTCTTG |  |
| oLVG029C | GGCGGCATGGATGAACTTTACAAATAGGTGTAAGGTGTATGGAA |  |
| oLVG029D | GGATTTCCTTACGCGAAATACGGGCGCTGATGTGTACTGGCC |  |
| oNLF264 | TTGTCCCGCACAAGGATCTGAAGAAGTATATGTTTTTGGTAATC |  |
| oNLF265 | ACCAAAAACATATACTTCTTCAGATCCTTGTGCGGGAC |  |
| oNLF266 | CTTCAACAATGCCAATTGAATTAAAAGTAGCACATGAAAAGTTGGATG |  |
| oNLF267 | TTTTCATGTGCTACTTTTAATTCAATTGGCATTGTTGAAG |  |
| oNLF032 | GCATAACCAAGCCTATGCCTACAGCAGCGCCCAATTATTTGTAC |  |
| oLM1 | TCGAGCACCACCACCACCAC |  |
| oLM2 | ACCTCCAATCTGTTCGCGGTG |  |
| oNLF001 | /5IRD700/CA AAACAGGATATGAAATAGTATTGGACG**A**GAGCTTTTTGGTGGCTTATACTATAG | EMSA Analysis |
| oTMN067 | /5IRD700/CA AAACAGGATATGAAATAGTATTGGACGAGAGCTTTTTGGTGGCTTATACTATAG |  |
| oTMN068 | /5IRD800/CTATAGTATAAGCCACCAAAAAGCTCTCGTCCAATACTATTTCATATCCTGTTTTG |  |
| oJRR271* | /5IRD800/rCrUrArUrArGrUrArUrArArGrCrCrArCrCrArArArArArGrCrUrCrUrCrGrUrCrCrArArUrArCrUrArUrUrUrCrArUrArUrCrCrUrGrUrUrUrUrG |  |

* Indicates RNA oligonucleotide5' infrared dye is denoted as 5IRD800 or 5IRD700 with excitation at 800 nm or 700 nm respectively.
